# Body Roundness Index and Body Shape Index as Predictors for All‐Cause Mortality Beyond Body Mass Index: Findings From a National Cohort Study

**DOI:** 10.1155/jobe/7923338

**Published:** 2026-01-31

**Authors:** Yuya Kimura, Norihiko Inoue, Hideo Yasunaga

**Affiliations:** ^1^ Department of Health Services Research, Graduate School of Medicine, The University of Tokyo, Tokyo, Japan, u-tokyo.ac.jp; ^2^ Clinical Research Center, NHO Tokyo National Hospital, Tokyo, Japan; ^3^ Institute of Clinical Epidemiology (iCE), Showa University, Tokyo, Japan, showa-u.ac.jp; ^4^ Department of Clinical Data Management and Research, Clinical Research Center, National Hospital Organization Headquarters, Tokyo, Japan; ^5^ Department of Health Policy and Informatics, Graduate School of Medical and Dental Sciences, Institute of Science Tokyo, Tokyo, Japan; ^6^ Department of Clinical Epidemiology and Health Economics, School of Public Health, The University of Tokyo, Tokyo, Japan, u-tokyo.ac.jp

**Keywords:** body mass index, mortality, obesity

## Abstract

**Objective:**

Body mass index (BMI) has been criticised for its inability to differentiate between fat and nonfat tissues and to reflect body fat distribution. Body Roundness Index (BRI) and A Body Shape Index (ABSI) are novel indices addressing these limitations, yet their mortality risk‐stratification utility remains understudied in Asian populations.

**Methods:**

In this retrospective cohort study using a nationally representative Japanese claims database (2014–2022), we analysed 778,812 individuals who underwent healthcare checkups. Anthropometric indices were categorised into five groups based on restricted cubic spline curve–derived cutoffs. Multivariate Cox proportional hazards models, adjusted for demographic factors, lifestyle variables and comorbidities, assessed associations between these categorical variables and all‐cause mortality.

**Results:**

Among participants (mean [standard deviation] age of 62.8 [9.6] years and 445,250 [57.2%] women), 14,690 deaths occurred during a median [interquartile range] follow‐up of 4.53 [3.28–6.23] years. While BMI and BRI showed U‐shaped relationships with all‐cause mortality, ABSI demonstrated a J‐shaped relationship. Significant differences in mortality risk compared with the reference category were observed in three categories for BMI, four categories for BRI and four categories for ABSI.

**Conclusions:**

BRI and ABSI identified mortality risk differences across more categories than BMI, indicating that these indices may provide additional insights beyond BMI for mortality risk assessment.

**Summary**
• What is already known about this subject?
◦ Body mass index (BMI) has been criticised for its inability to account for variations in body fat percentage and distribution, as these can significantly affect mortality risk.
◦ Body Roundness Index (BRI) and A Body Shape Index (ABSI) have recently emerged as novel anthropometric indices that may address these fundamental limitations of BMI in terms of assessing body composition.
• What are the new findings in your manuscript?
◦ In this large‐scale Japanese cohort study, when three anthropometric indices were categorised into five groups in the same manner, significant mortality risk differences compared with the reference category were observed in three categories for BMI, four categories for BRI and four categories for ABSI.
• How might your results change the direction of research or the focus of clinical practice?
◦ This study provides reference values for anthropometric indices in Asian populations and suggests the potential benefits of considering novel anthropometric indices for mortality risk assessment.
◦ These findings may contribute to the development of more comprehensive approaches to body composition assessment and obesity management.

## 1. Introduction

Body mass index (BMI) has been widely used to estimate body fat, although it was originally designed to assess population‐level health risks rather than individual ones [1, 2]. However, BMI faces two major criticisms: It fails to differentiate between fat and nonfat mass (e.g., muscle) [3–6], and it does not account for fat distribution [7]. The first is illustrated by the fact that two individuals with identical BMIs can have vastly different body compositions: One might be muscular, with a figure such as that of Arnold Schwarzenegger, whereas the other might be out of shape [2]. Therefore, assuming an equal mortality risk for these individuals based on BMI alone is likely inaccurate. Recent research has indicated that the visceral‐to‐subcutaneous fat area ratio is more consistently associated with all‐cause mortality than BMI, suggesting that fat distribution may be more crucial than overall body fat in terms of assessing mortality risk [8]. To address these limitations, novel anthropometric indices incorporating waist circumference (WC) have been proposed, including the Body Roundness Index (BRI) and A Body Shape Index (ABSI) [7, 9]. A recent study using nationally representative US data from the National Health and Nutrition Examination Survey revealed that, while BRI and BMI showed U‐shaped associations with all‐cause mortality, BRI demonstrated narrower confidence intervals, suggesting that it may offer more precise mortality risk estimates than BMI [2, 10]. Studies on Asian individuals with Type 2 diabetes, as well as a similar cohort from 10 European countries, indicated that combining ABSI with BMI could improve mortality estimates compared with using BMI alone [11, 12]. However, data regarding the association between BRI or ABSI and all‐cause mortality in nationally representative Asian populations remain limited. Furthermore, it remains unclear whether BRI or ABSI can provide more detailed risk stratification within BMI categories, given that individuals in the same BMI category can have significantly different mortality risks.

We hypothesised that, even in Asian populations, BRI and ABSI would yield narrower confidence intervals for mortality hazard ratios (HRs) than BMI or that the number of categories showing significant differences in mortality risk compared with the reference category would be greater. We further hypothesised that these indices would allow more detailed stratification of mortality risk within BMI categories. Therefore, this study aimed to investigate the associations of BMI, BRI and ABSI with all‐cause mortality and to clarify the extent of risk stratification that these indices can provide within each BMI category.

## 2. Materials and Methods

### 2.1. Data Source

We used the DeSC database (DeSC Healthcare Inc., Tokyo, Japan), which constitutes a nationally representative database of health insurance claims in Japan [13]. This comprehensive database collects information from three types of insurers: (1) the National Health Insurance for nonemployees and individual proprietors; (2) health insurance for employees of large companies; and (3) the Advanced Elderly Medical Service System for those aged ≥ 75 years and those aged 65–75 years with certain disorders. This database includes approximately 12.5 million individuals with an age distribution that closely mirrors that of the overall Japanese population [13]. One notable feature of this database is the availability of accurate mortality data for individuals covered by the National Health Insurance and the Advanced Elderly Medical Service System. The database contains unique identifiers, demographic information, and detailed records of diagnoses (coded according to the International Classification of Diseases, 10^th^ Revision [ICD‐10]), examinations, and treatments, in inpatient and outpatient settings. The database also contains healthcare checkup data for individuals aged ≥ 40 years. This additional information includes demographic characteristics (height, weight and WC), smoking and drinking statuses, sleep patterns, physical activity levels and blood test results. The measurements of height, weight and WC were performed by trained healthcare professionals (e.g., nurses). Height and weight were measured using a calibrated stadiometer and scale, with participants standing barefoot and wearing light clothing. WC was measured using a nonstretchable tape while the participant was standing upright; the tape was placed at the level of the umbilicus following normal expiration. For individuals with severe obesity in whom the umbilicus was not a suitable landmark, WC was measured at the midpoint between the lower rib margin and the iliac crest. These procedures follow the standardised methods specified for Japanese health checkups.

### 2.2. Study Design and Population

The study period spanned from 1 April 2014 to 30 November 2022. We followed individuals from their initial healthcare checkup date (designated as the ‘index date’), which occurred at least three months after their registration in the database. Follow‐up was continued until death, withdrawal from insurance or 30 November 2022, whichever occurred first. The three‐month buffer period was necessary to accurately assess comorbidity status.

We included individuals enrolled in either the National Health Insurance or the Advanced Elderly Service System who had undergone a health checkup to determine their index date. We did not include employees enrolled in health insurance for large companies because their mortality data were unavailable. We excluded individuals with follow‐up periods of < 2 years to avoid the potential effects of reverse causality; cases in which height, weight or WC data were missing from the index‐date health checkup; those with diagnostic codes suggesting pregnancy (ICD‐10 Codes O00–O99) within three months preceding the index date, as pregnancy can temporarily increase WC and those with missing data regarding smoking and drinking statuses, sleep patterns or physical activity levels.

### 2.3. Calculation of BRI and ABSI

BRI and ABSI were calculated on the basis of their definitions according to the following formulae [7, 9]:

(1) BRI=364.2365.5−×1− WCcm 2π/ 0.5×heightcm2,ABSI=WC / BMIkg/m223/×heightcm12/.

### 2.4. Outcome

All‐cause mortality was defined as the primary outcome. Specific causes of death could not be identified, owing to limitations in the data collected by the insurers. Thus, our analysis focused on all‐cause mortality without distinguishing between different causes of death.

### 2.5. Covariates

The covariates included age, sex, smoking and drinking statuses, sleep patterns, physical activity levels, Type 2 diabetes and cardiovascular diseases as comorbidities. These comorbidities were identified using the corresponding ICD‐10 codes (E11 for Type 2 diabetes and I21, I60, I61 and I63 for cardiovascular diseases) [14–16] recorded within three months prior to the index date.

### 2.6. Primary Analysis

We constructed restricted cubic spline (RCS) curves with four knots to assess nonlinearity between the three anthropometric indices and all‐cause mortality, as well as to determine cutoff points for each. Based on these cutoff points, each anthropometric index was divided into five categories, from Q1 (the lowest) to Q5 (the highest). Despite the existing World Health Organization cutoffs for BMI [17], we opted to establish our own cutoffs for all indices to ensure fairness and consistency. Using a Cox proportional hazards model (time scale: days), we calculated the adjusted HRs for each anthropometric index category using three models: (1) adjusted for age and sex, (2) further adjusted for lifestyle factors (smoking, drinking, sleep and physical activity) and (3) additionally adjusted for comorbidities (Type 2 diabetes and cardiovascular diseases). All analyses were performed using R (Version 4.3.1).

### 2.7. Sensitivity Analysis

In our primary analysis, we assumed that data from the index date remained constant throughout the observational period. To test the robustness of our primary analysis findings, we conducted a sensitivity analysis using a time‐varying survival analysis, in which the values of BMI, BRI and ABSI were updated at each subsequent healthcare checkup. These updated values replaced the previous ones and were used as time‐varying covariates in the Cox model throughout the follow‐up period. However, to avoid potential reverse causality, we did not use healthcare checkup data from the 2 years preceding the end of the observation period. In our sensitivity analysis, we used the same cutoff values for the anthropometric indices as those used in the primary analysis.

### 2.8. Exploratory Analysis

We investigated how each anthropometric index subcategory differentiated mortality risk in each BMI category (BMI Q1–Q5). This exploratory analysis aimed to assess whether BRI and ABSI, which reflect body fat percentage and distribution, could further differentiate mortality risk within BMI‐defined categories that do not capture these characteristics. Specifically, within each BMI category (Q1–Q5), we generated RCS curves with four knots to determine cutoff points for the three indices. Then, each index was categorised into five subgroups (QNa–QNe), where ‘N’ represents the original BMI category number. For example, within the lowest BMI category (Q1), five subcategories ranging from Q1a (lowest) to Q1e (highest) were created. We calculated HRs for each subgroup compared to the corresponding reference, following the same methodology as in the primary analysis.

### 2.9. Subgroup Analysis

Given the potential differences in estimates between men and women, we conducted subgroup analyses stratified by sex, following the same methodology as in the primary analysis.

### 2.10. Ethics Approval

This study was approved by the institutional review board of the University of Tokyo (approval number: 2021010NI [23 April 2021]) and was performed in accordance with the tenets of the Declaration of Helsinki. The requirement for written consent was waived, owing to the retrospective study design and use of anonymised patient data.

## 3. Results

### 3.1. Baseline Characteristics

**FIGURE 1:**
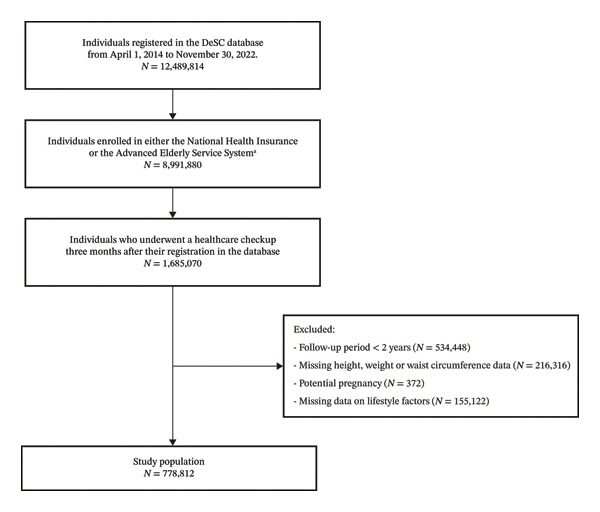
Flowchart of patient inclusion. ^a^Mortality data were available for those individuals who were enrolled in either the National Health Insurance or the Advanced Elderly Service System.

A total of 778,812 individuals (445,250 [57.2%] women) with a mean (standard deviation) age of 62.8 (9.6) years were enrolled (Table 1 and Figure 1). Compared with the survivors, those who died were generally older and predominantly male. Regarding lifestyle factors, the deceased individuals were more likely to have been smokers, daily drinkers and physically inactive than survivors (Table 1). They also had a higher prevalence of Type 2 diabetes and cardiovascular diseases. ABSI was higher in the deceased individuals than in survivors, whereas BMI and BRI showed no difference between the groups (absolute standard difference, ≤ 0.10). While BRI demonstrated a strong correlation with BMI (*γ* = 0.81), ABSI showed little correlation with BMI (*γ* = −0.04).

**TABLE 1:** Individual patient characteristics.

	Total number of individuals	Number of individuals who survived	Number of individuals who died	ASD
Total	778,812 (100.0%)	764,122 (100.0%)	14,690 (100.0%)	
Age (y)	62.8 (9.6)	62.7 (9.6)	68.0 (8.5)	0.59
Women	445,250 (57.2%)	440,106 (57.6%)	5144 (35.0)	0.47

**Anthropometric indices**				

BMI	23.00 (3.55)	23.00 (3.55)	22.92 (3.77)	0.02
BRI	3.86 (1.27)	3.86 (1.27)	3.93 (1.35)	0.06
ABSI	0.82 (0.05)	0.82 (0.05)	0.83 (0.05)	0.21

**Lifestyle factors**				

*Smoking status*				0.23
Current	118,614 (15.2%)	115,102 (15.1%)	3512 (23.9%)	
Past/never	660,198 (84.8%)	649,020 (84.9%)	11,178 (76.1%)	

*Drinking status*				0.16
Everyday	183,385 (23.5%)	178,978 (23.4%)	4407 (30.0%)	
Sometimes	163,096 (20.9%)	160,603 (21.0%)	2493 (17.0%)	
Seldom/never	432,331 (55.5%)	424,541 (55.6%)	7790 (53.0%)	

*Sleep pattern*				0.04
Adequate	562,634 (72.2%)	551,787 (72.2%)	10,847 (73.8%)	
Inadequate	216,178 (27.8%)	212,335 (27.8%)	3843 (26.2%)	
Physical activity level				0.10
High^a^	392,601 (50.4%)	385,879 (50.5%)	6722 (45.8%)	
Low	386,211 (49.6%)	378,243 (49.5%)	7968 (54.2%)	

**Comorbidity**				

Type 2 diabetes	46,967 (6.0%)	45,407 (5.9%)	1560 (10.6%)	0.17
Cardiovascular diseases	36,062 (4.6%)	34,645 (4.5%)	1417 (9.6%)	0.10

*Note:* Continuous variables are presented as means (standard deviations), while categorical ones are presented as numbers (%).

Abbreviations: ABSI, A Body Shape Index; ASD, absolute standard difference; BMI, body mass index; BRI, Body Roundness Index.

^a^‘High physical activity’ was defined as engaging in physical activities equivalent to walking for at least 1 hour per day.

### 3.2. Associations of BMI, BRI and ABSI With All‐Cause Mortality

During a median [interquartile range] follow‐up period of 4.53 [3.28–6.23] years, 14,690 deaths occurred. Figure 2 shows the RCS curves for the association between the anthropometric indices and all‐cause mortality. BMI and BRI demonstrated U‐shaped relationships with all‐cause mortality, with BRI showing a more pronounced U shape than BMI. By contrast, ABSI showed a J‐shaped relationship with all‐cause mortality. The range of risk estimates was comparable between BMI and BRI, whereas ABSI showed a slightly narrower range.

FIGURE 2Association between anthropometric indices and all‐cause mortality risk after adjustment. The solid curves represent the point estimates for the association between anthropometric indices and all‐cause mortality, with the shaded areas indicating 95% confidence intervals. To avoid extrapolation, the curves were restricted to the range between the 1^st^ and 99^th^ percentiles for each anthropometric index. Point estimates and 95% confidence intervals for these associations at the 1^st^ and 99^th^ percentiles are presented. The nadirs of the curves for body mass index, Body Roundness Index and A Body Shape Index are observed at values of 23.6, 3.58 and 0.71, corresponding to minimum hazard ratios (95% confidence intervals) of 0.99 (0.97–1.00), 1.00 (0.99–1.00) and 0.84 (0.75–0.93), respectively. (a) Body mass index. (b) Body Roundness Index. (c) A Body Shape Index.

**Figure:**
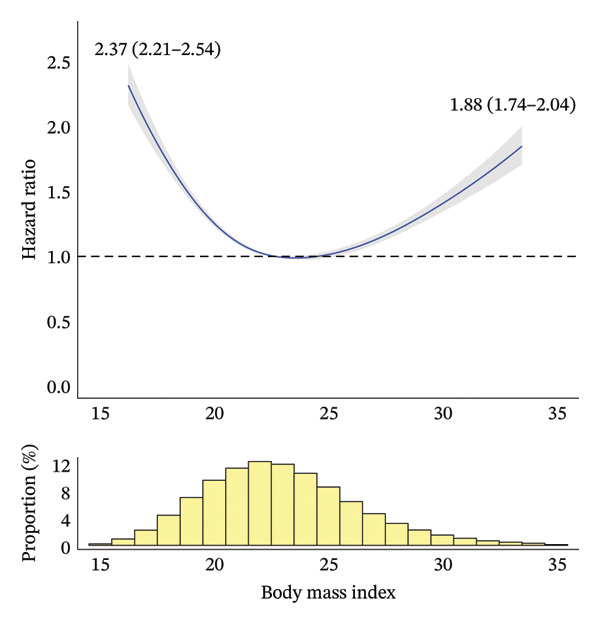
(a)

**Figure:**
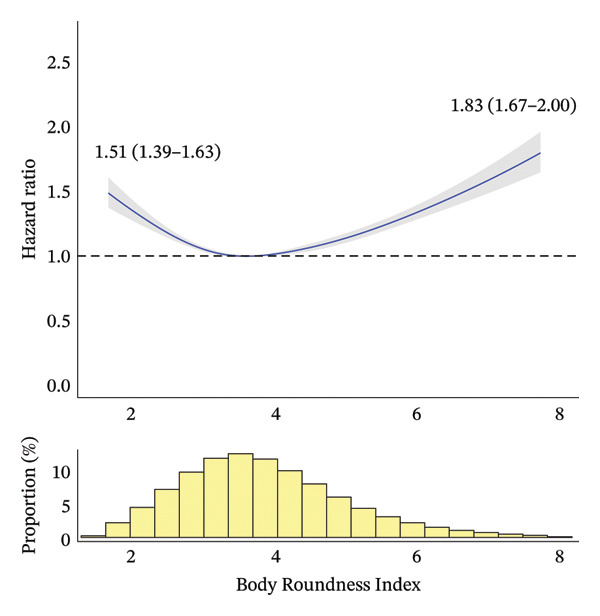
(b)

**Figure:**
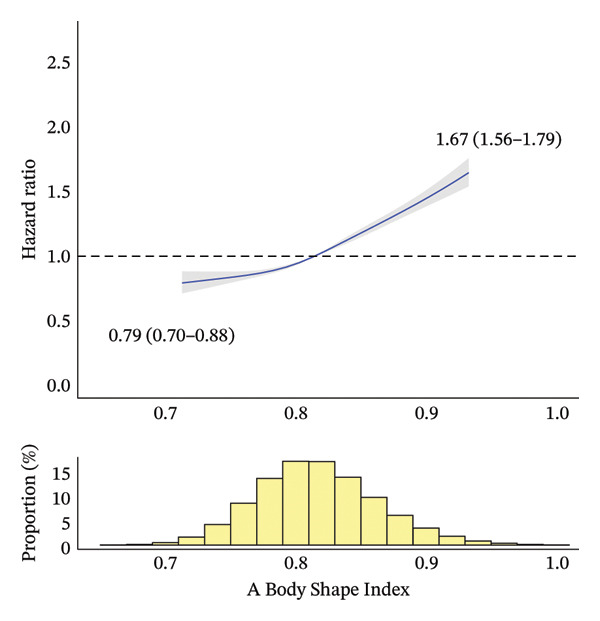
(c)

Table 2 presents HRs for all‐cause mortality by anthropometric index categories, before and after covariate adjustment. For BMI, after adjustment and when compared with Q3 (the reference category), Q1, Q2 and Q5 all showed significantly higher mortality rates. Specifically, after full adjustment, Q1 and Q5 were associated with 83% and 26% higher mortality rates, respectively, than Q3. For BRI, after adjustment and when compared with Q3, all of the other categories showed significantly higher mortality rates. Specifically, after full adjustment and when compared with Q3, Q1 and Q5 were associated with 34% and 37% higher mortality rates, respectively. For ABSI, after adjustment and when compared with Q3, Q1 and Q2 showed significantly lower mortality rates, whereas Q4 and Q5 showed significantly higher ones. Specifically, after full adjustment, Q1 was associated with 13% lower mortality than Q3, whereas Q5 showed 47% higher mortality. Notably, while the adjusted analyses results (including RCS curves) were largely consistent with the unadjusted results for BMI (i.e., a U‐shaped association) and ABSI (a positive relationship), BRI showed different patterns: a U‐shaped association after adjustment (and in our RCS curves) versus a positive relationship before adjustment.

**TABLE 2:** Hazard ratios of all‐cause mortality according to the quartiles of the three anthropometric indices.

**Hazard ratio (95% confidence intervals)**					
**Body mass index**					
	**Q1: −17.93**	**Q2: 17.94–21.43**	**Q3: 21.44–23.93**	**Q4: 23.94–29.03**	**Q5: 29.04-**

Without adjustment	1.35 [1.25–1.45]	0.98 [0.94–1.03]	1 [Reference]	1.05 [1.00–1.09]	1.06 [0.97–1.15]
Adjusted for age and sex	1.93 [1.79–2.08]	1.23 [1.18–1.28]	1 [Reference]	1.01 [0.97–1.06]	1.34 [1.23–1.46]
Partially adjusted^a^	1.81 [1.69–1.95]	1.20 [1.15–1.26]	1 [Reference]	1.01 [0.97–1.05]	1.31 [1.20–1.42]
Fully adjusted^b^	1.83 [1.71–1.97]	1.21 [1.16–1.27]	1 [Reference]	1.00 [0.95–1.04]	1.26 [1.16–1.37]

**Body Roundness Index**					
	**Q1: −2.14**	**Q2: 2.15–3.28**	**Q3: 3.29–4.14**	**Q4: 4.15–5.99**	**Q5: 6.00-**

Without adjustment	0.94 [0.86–1.02]	0.94 [0.90–0.98]	1 [Reference]	1.07 [1.03–1.12]	1.26 [1.17–1.36]
Adjusted for age and sex	1.36 [1.25–1.48]	1.11 [1.06–1.16]	1 [Reference]	1.11 [1.07–1.16]	1.49 [1.38–1.60]
Partially adjusted^a^	1.32 [1.22–1.43]	1.10 [1.06–1.15]	1 [Reference]	1.10 [1.05–1.15]	1.42 [1.32–1.53]
Fully adjusted^b^	1.34 [1.24–1.45]	1.11 [1.07–1.16]	1 [Reference]	1.08 [1.04–1.13]	1.37 [1.27–1.48]

**A Body Shape Index**					
	**Q1: −0.74**	**Q2: 0.75–0.79**	**Q3: 0.80–0.83**	**Q4: 0.84–0.89**	**Q5: 0.90-**

Without adjustment	0.52 [0.47–0.58]	0.77 [0.74–0.81]	1 [Reference]	1.14 [1.10–1.19]	1.37 [1.27–1.47]
Adjusted for age and sex	0.84 [0.76–0.93]	0.87 [0.83–0.91]	1 [Reference]	1.21 [1.16–1.26]	1.55 [1.44–1.67]
Partially adjusted^a^	0.86 [0.78–0.96]	0.89 [0.85–0.93]	1 [Reference]	1.18 [1.13–1.23]	1.47 [1.37–1.59]
Fully adjusted^b^	0.87 [0.79–0.97]	0.90 [0.86–0.94]	1 [Reference]	1.18 [1.13–1.22]	1.47 [1.36–1.58]

^a^Adjusted for age, sex and lifestyle factors (smoking and drinking statuses, sleep patterns and physical activity level).

^b^Adjusted for age, sex, lifestyle factors and comorbidities (Type 2 diabetes and cardiovascular diseases).

### 3.3. Sensitivity Analysis

Table S1 (Online Supporting File S1) shows the HRs for all‐cause mortality according to the anthropometric index categories before and after covariate adjustment in our time‐varying survival analysis. While the associations were slightly attenuated compared with those observed in our primary analyses, the overall patterns remained consistent.

### 3.4. Exploratory Analysis

In our BMI‐stratified analyses, BMI exhibited different mortality relationships across categories: negative relationships in Q1 and Q2, a constant pattern in Q3 and positive relationships in Q4 and Q5 (with a J‐shaped pattern in Q5; Online Supporting File S1, Figure S1). By contrast, BRI and ABSI consistently demonstrated positive relationships across all BMI categories, with ABSI showing a J‐shaped pattern in Q5. When examining the distributional patterns compared to the overall population, BRI showed progressive shifts: significant leftward shifts in Q1 and Q2, a slight leftward shift in Q3, a slight rightward shift in Q4 and a pronounced rightward shift in Q5. By contrast, the ABSI distribution remained consistent with the overall population distribution across all BMI categories (Figure 2; Online Supporting File S1, Figure S1). When comparing mortality risks across subcategories to QNc (the reference category) within each BMI category (Q1–Q5), the patterns varied according to the BMI category (Online Supporting File S1, Table S2). In BMI Q1 (lowest), significant mortality differences were observed in two, none and three subcategories for BMI, BRI and ABSI, respectively. In BMI Q2 (second lowest), the corresponding numbers were two, three and three; in BMI Q3 (middle), none, four and two; in BMI Q4 (second largest), one, four and four; and in BMI Q5 (largest), one, two and one, respectively. Detailed results from these exploratory analyses are provided in Online Supporting File S1.

### 3.5. Subgroup Analysis

Table S3 (Online Supporting File S1) presents the HRs for all‐cause mortality according to anthropometric index categories in the subgroup analysis stratified by sex. While the associations were slightly different between sexes, the overall patterns remained consistent.

## 4. Discussion

### 4.1. Summary of the Findings

Our analysis of this nationally representative Japanese cohort revealed several key findings across overall and BMI‐stratified analyses. In the overall population, BMI and BRI demonstrated U‐shaped associations with all‐cause mortality, with BRI showing a more pronounced relationship, whereas ABSI exhibited a J‐shaped association. The estimated risk ranges were similar for BMI and BRI, with ABSI showing a slightly narrower range. When comparing mortality risks across categories to Q3 (the reference category), significant differences were found in three of four categories for BMI and in all categories for BRI and ABSI. Notably, BMI and ABSI maintained consistent mortality risk patterns before and after adjustment, whereas BRI showed a distinct pattern in the adjusted and unadjusted analyses. In our BMI‐stratified analyses, BMI exhibited negative relationships in Q1 and Q2, a constant pattern in Q3 and positive relationships in Q4 and Q5. By contrast, BRI and ABSI consistently demonstrated positive relationships across all BMI categories (Q1–Q5).

### 4.2. Comparison With Previous Studies

In our large‐scale, general population cohort of 778,812 Asian adults, we observed a U‐shaped association between BRI and all‐cause mortality and a J‐shaped association between ABSI and all‐cause mortality. These findings are consistent with those of previous research: a U‐shaped association between BRI and all‐cause mortality in a national cohort of 32,995 US adults [10], a J‐shaped association of ABSI among 352,985 participants in the UK Biobank [12] and U‐shaped (BRI) and J‐shaped (ABSI) associations in 11,872 Taiwanese patients with diabetes [11]. This consistency reinforces the generalisability of the mortality association patterns of BRI and ABSI to broader Asian populations. Notably, the cutoff values of the anthropometric indices in our study were lower than those reported in the US study on which our analysis plan was based [10]. This difference likely reflects variations in body composition across populations: Most participants in our study were of Asian ethnicity, who are generally characterised by smaller body frames and lower muscle mass, whereas the US study primarily included Western populations with larger body builds. Therefore, the cutoffs identified in our study may serve as reference values more suitable for Asian than for Western populations.

In our study, while the risk estimate ranges were similar between BMI and BRI, ABSI showed narrower ranges than BMI, and BRI and ABSI identified mortality risk differences across more categories than BMI. This suggests that BRI and ABSI may provide additional insights beyond BMI for mortality risk assessment. However, given that BRI showed inconsistent mortality risk patterns between the adjusted and unadjusted analyses, whereas ABSI showed consistent patterns, ABSI may be considered a more clinically intuitive and practically usable index than BRI.

Our BMI‐stratified analyses indicated that, within each BMI category, BRI or ABSI identified a greater number of subcategories with significant mortality differences compared with the reference subcategory than BMI, although the patterns varied across BMI categories. This was particularly evident in the BMI Q3 category, where BMI‐based subcategorisation showed no significant risk differences between groups, whereas BRI‐ and ABSI‐based subcategorisation revealed significant stepwise differences in risk. ABSI was originally designed to be independent of BMI [7], and our study confirmed this independence with correlation coefficients near zero between ABSI and BMI, even in our Asian cohort. This independence was further reflected in the consistency of the ABSI distributions across BMI categories, which closely matched the overall population distribution. These findings suggest that ABSI may be the preferred index for additional risk stratification within BMI categories.

### 4.3. Practical Challenges and Future Directions

While our findings suggest the possible advantages of BRI and ABSI over BMI for mortality risk stratification, these indices capture different aspects of body composition. BMI is derived solely from height and weight and does not distinguish between fat and lean mass. Conversely, BRI incorporates WC relative to height, serving as a better proxy for central adiposity, whereas ABSI combines BMI, WC and height to emphasise body shape and fat distribution relative to body size [7, 9]. Such mechanistic differences may explain the distinct mortality associations across the indices. Nevertheless, the practical implementation of these novel indices faces challenges. BMI has long been used in many clinical settings and is much simpler to calculate than the more complex computations required for BRI and ABSI. Furthermore, the future of anthropometric risk assessment may extend beyond all of these indices. With advancing technology that has enabled the collection of multiple body composition parameters, as well as the recent development of machine learning algorithms, more accurate mortality risk predictions may become feasible. At present, recognising that BMI should not be considered the sole anthropometric index for mortality risk stratification while continuing to accumulate evidence on the utility of alternative indices seems to be the most practical approach.

### 4.4. Strengths and Limitations

To our knowledge, this is the largest study to date to investigate the clinical utility of BRI and ABSI as mortality risk stratification tools compared to BMI in an Asian population. Furthermore, we clarified the utilities of BRI and ABSI for mortality risk stratification within each BMI category, highlighting the potential limitations of using BMI as a sole indicator. However, this study has some limitations. First, as most of the population in Japan is of Asian ethnicity and the data source we used does not include information regarding ethnicity, we could not consider ethnic variations. Second, owing to the absence of cause‐specific mortality data, we could not analyse disease‐specific mortality patterns. Third, the median observational period of 4.53 years was short. Fourth, some selection bias may exist. For example, to investigate the association between anthropometric indices and mortality, we had to exclude data from individuals without anthropometric measurements and from employees enrolled in health insurance for large companies (for whom mortality data are unavailable). Last, socioeconomic status variables, such as education and income, were not available in our database and could not be adjusted for. This may have led to residual confounding factors.

## 5. Conclusions

In this nationwide Japanese cohort, the novel anthropometric indices BRI and ABSI, which incorporate WC measurements, identified mortality risk differences across more categories than BMI, both overall and within different BMI categories. Therefore, our findings caution against relying solely on BMI for mortality risk assessments.

## Author Contributions

Yuya Kimura and Norihiko Inoue designed the study. Yuya Kimura performed the analyses and wrote the first version of the manuscript. Yuya Kimura, Norihiko Inoue and Hideo Yasunaga interpreted the results, critically revised the manuscript and approved its final submitted version. Yuya Kimura is the guarantor.

## Funding

This work was supported by a grant from the Ministry of Health, Labor and Welfare of Japan (grant number 23AA2003).

## Disclosure

The funding body had no role in the design of the study, collection, analysis or interpretation of the data or writing of the manuscript.

## Conflicts of Interest

The authors declare no conflicts of interest.

## Supporting Information

Detailed description about the exploratory analysis. Table S1. Hazard ratios of all‐cause mortality according to anthropometric index quantiles in our primary and sensitivity analyses. Figure S1. Association between anthropometric indices and all‐cause mortality risk after adjustment within body mass index categories (Q1–Q5). Table S2. Hazard ratios of all‐cause mortality according to anthropometric index subcategories within individual body mass index categories (Q1–Q5).

## Supporting information


**Supporting Information** Additional supporting information can be found online in the Supporting Information section.

## Data Availability

The datasets analysed in this study are commercially available from DeSC Healthcare, Inc.
