# Supplementary material for: Body Roundness Index and Body Shape Index as Predictors for All‐Cause Mortality Beyond Body Mass Index: Findings From a National Cohort Study
Source: J Obes. 2026 Jan 31;2026:7923338. doi: 10.1155/jobe/7923338 (PMC12860135; doi:10.1155/jobe/7923338)
Supplement: Supplementary file 1 — Supporting Information Additional supporting information can be found online in the Supporting Information section. [file JOBE-2026-7923338-s001.zip › clean_manuscript_supplement_20250827.docx]

**Body Roundness Index and Body Shape Index as Predictors for All-Cause Mortality Beyond Body Mass Index: Findings from a National Cohort Study**

Yuya Kimura, MD MPH^1,2^; Norihiko Inoue, MD PhD^3,4,5^; Hideo Yasunaga, MD PhD^6^

^1^Department of Health Services Research, Graduate School of Medicine, The University of Tokyo, Tokyo, Japan

^2^Clinical Research Center, NHO Tokyo National Hospital, Tokyo, Japan

^3^Department of Health Policy and Informatics, Graduate School of Medical and Dental Sciences, Institute of Science Tokyo, Tokyo, Japan

^4^Department of Clinical Data Management and Research, Clinical Research Center, National Hospital Organization Headquarters, Tokyo, Japan

^5^Institute of Clinical Epidemiology (iCE), Showa University, Tokyo, Japan

^6^Department of Clinical Epidemiology and Health Economics, School of Public Health, The University of Tokyo, Tokyo, Japan

CONTACT INFO:

Yuya Kimura (Corresponding author)

Department of Health Services Research, Graduate School of Medicine, The University of Tokyo, Tokyo, Japan; Clinical Research Center, National Hospital Organization Tokyo Hospital, Tokyo, Japan

E-mail: yuk.close.to.wrd.34@gmail.com

***Exploratory analysis (detailed description)***

In the lowest BMI category (Q1), BMI showed a reverse J-shaped relationship with all-cause mortality, whereas BRI and ABSI exhibited positive relationships (Figure S1). Compared to the distribution in the overall population, the BRI distribution among individuals in BMI Q1 was shifted leftward, whereas the ABSI distribution remained similar (Figures 2 and S1). After adjusting for BMI and conducting comparisons with Q1c (the reference category), Q1a and Q1b showed significantly higher mortality rates (Table S2). For BRI, following adjustment and comparison with Q1c, mortality risks across the other subcategories showed no significant differences. After adjusting for ABSI and conducting a comparison with Q1c, Q1b showed significantly lower mortality, whereas Q1d and Q1e showed significantly higher mortality.

In the second lowest BMI category (Q2), BMI showed a reverse J-shaped relationship with all-cause mortality, whereas BRI and ABSI exhibited positive relationships (Figure S1). Compared to the distribution in the overall population, the BRI distribution among individuals in BMI Q2 showed a slight leftward shift, whereas the ABSI distribution remained similar (Figures 2 and S1). For BMI, following adjustment and conducting a comparison with Q2c (the reference category), Q2a and Q2b showed significantly higher mortality rates (Table S2). For BRI, following adjustment and a comparison with Q2c, Q2a and Q2b showed significantly lower mortality rates, while Q2d and Q2e showed significantly higher ones. For ABSI, after adjustment and a comparison with Q2c, Q2b showed a significantly lower mortality rate, whereas Q2d and Q2e showed significantly higher ones.

In the middle BMI category (Q3), BMI showed a consistent relationship with all-cause mortality, whereas BRI and ABSI showed J-shaped relationships (Figure S1). Compared to the distribution in the overall population, the BRI distribution among individuals in BMI Q3 showed a slight leftward shift, whereas the ABSI distribution remained similar (Figures 2 and S1). For BMI, following adjustment and comparison with Q3c (the reference category), the mortality risks across the other subcategories showed no significant differences (Table S2). For BRI, following adjustment and a comparison with Q3c, Q3a and Q3b showed significantly lower mortality rates, while Q3d and Q3e showed significantly higher ones. For ABSI, following adjustment and comparison with Q3c, Q3b showed a significantly lower mortality rate, whereas Q3d and Q3e showed significantly higher ones.

In the second highest category (Q4), BMI demonstrated a J-shaped relationship with all-cause mortality, whereas BRI and ABSI showed positive linear relationships (Figure S1). Compared to the distribution in the overall population, the BRI distribution among individuals in BMI Q4 showed a slight rightward shift, whereas that for ABSI remained similar (Figures 2 and S1). For BMI, following adjustment and comparison with Q4c (the reference category), only Q4e had a significantly higher mortality rate (Table S2). For BRI, following adjustment and comparison with Q4c, Q4a and Q4b showed significantly lower mortality rates, whereas Q4d and Q4e showed significantly higher ones. For ABSI, following adjustment and comparison with Q4c, Q4a and Q4b showed significantly lower mortality rates, whereas Q4d and Q4e showed significantly higher ones.

In the highest category (Q5), BMI showed a J-shaped relationship with all-cause mortality, BRI showed a positive linear relationship, and ABSI exhibited a sigmoidal relationship (Figure S1). Compared to the distribution in the overall population, the BRI distribution among individuals with BMI Q5 was shifted rightward, whereas the ABSI distribution remained similar (Figures 2 and S1). For BMI, following adjustment and comparison with Q5c (the reference category), only Q5d showed a significantly higher mortality rate (Table S2). For BRI, following adjustment and comparison with Q5c, Q5a and Q5b showed significantly lower mortality rates. For ABSI, following adjustment and comparison with Q5c, Q5b showed a significantly lower mortality rate, whereas Q5d showed a significantly higher one.

**Table S1. Hazard ratios of all-cause mortality according to anthropometric index quantiles in our primary and sensitivity analyses**

| Hazard ratio (95% confidence intervals)^a^ | | | | | |  |
| --- | --- | --- | --- | --- | --- | --- |
| **Body mass index** | | | | | |  |
|  | **Q1: –17.93** | **Q2: 17.94–21.43** | **Q3: 21.44–23.93** | **Q4: 23.94–29.03** | **Q5: 29.04–** |  |
| Primary analysis | | 1.83 [1.71–1.97] | 1.21 [1.16–1.27] | 1 [Reference] | 1.00 [0.95–1.04] | 1.26 [1.16–1.37] |
| Sensitivity analysis | | 1.29 [1.19–1.39] | 1.04 [0.99–1.09] | 1 [Reference] | 0.99 [0.95–1.04] | 1.21 [1.12–1.30] |
| **Body roundness index** | | | | | | |
|  | | **Q1: –2.14** | **Q2: 2.15–3.28** | **Q3: 3.29–4.14** | **Q4: 4.15–5.99** | **Q5: 6.00–** |
| Primary analysis | | 1.34 [1.24–1.45] | 1.11 [1.07–1.16] | 1 [Reference] | 1.08 [1.04–1.13] | 1.37 [1.27–1.48] |
| Sensitivity analysis | | 1.16 [1.07–1.26] | 1.07 [1.02–1.12] | 1 [Reference] | 1.05 [1.01–1.10] | 1.25 [1.16–1.35] |
| **A body shape index** | | | | | | |
|  | | **Q1: –0.74** | **Q2: 0.75–0.79** | **Q3: 0.80–0.83** | **Q4: 0.84–0.89** | **Q5: 0.90–** |
| Primary analysis | | 0.87 [0.79–0.97] | 0.90 [0.86–0.94] | 1 [Reference] | 1.18 [1.13–1.22] | 1.47 [1.36–1.58] |
| Sensitivity analysis | | 0.94 [0.85–1.04] | 0.94 [0.90–0.98] | 1 [Reference] | 1.06 [1.01–1.10] | 1.15 [1.06–1.24] |

a. Adjusted for age, sex, lifestyle factors, and comorbidities (type 2 diabetes and cardiovascular diseases).

**Figure S1. Association between anthropometric indices and all-cause mortality risk after adjustment within body mass index categories (Q1–Q5)**

**
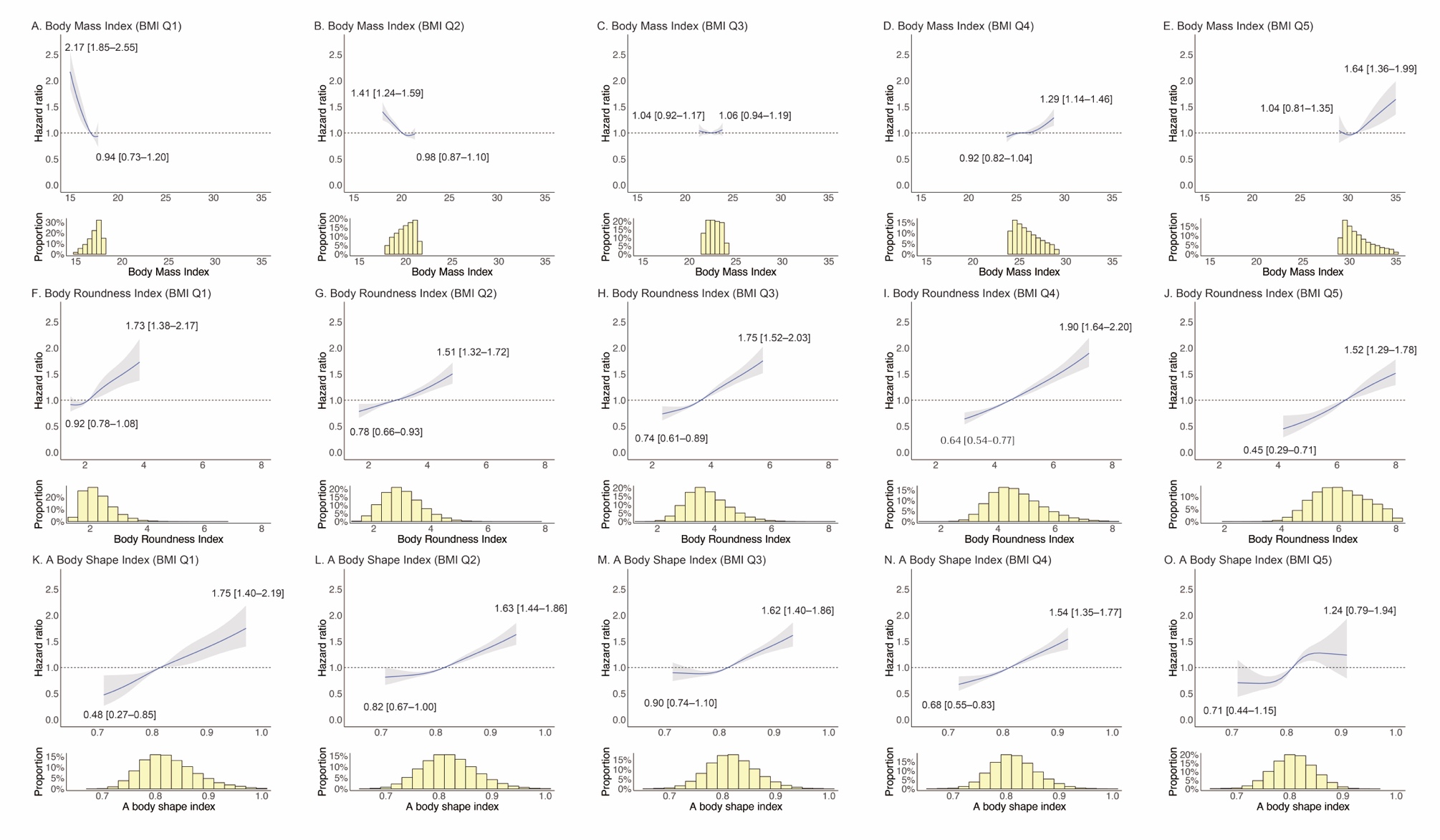
**

**Table S2. Hazard ratios of all-cause mortality according to anthropometric index subcategories within individual body mass index categories (Q1–Q5)**

| Hazard ratio (95% confidence intervals)^a^ | | | | | |  |  |  |
| --- | --- | --- | --- | --- | --- | --- | --- | --- |
| **In the lowest BMI category (Q1)** | | | | | |  |  |  |
| **BMI** | **Q1a: –15.58** | **Q1b: 15.59–16.86** | **Q1c: 16.87–17.45** | **Q1d: 17.46–17.87** | **Q1e: 17.88–17.93** |  |  |  |
|  | 1.89 [1.42–2.51] | 1.29 [1.09–1.53] | 1 [Reference] | 0.92 [0.77–1.10] | 0.92 [0.66–1.30] |  |  |  |
| **BRI** | **Q1a: –1.60** | **Q1b: 1.61–1.99** | **Q1c: 2.00–2.39** | **Q1d: 2.40–3.27** | **Q1e: 3.28–** |  |  |  |
|  | 0.75 [0.53–1.06] | 0.86 [0.72–1.02] | 1 [Reference] | 1.06 [0.89–1.27] | 1.30 [0.97–1.74] |  |  |  |
| **ABSI** | **Q1a: –0.75** | **Q1b: 0.76–0.80** | **Q1c: 0.81–0.84** | **Q1d: 0.85–0.91** | **Q1e: 0.92–** |  |  |  |
|  | 0.65 [0.41–1.03] | 0.83 [0.69–1.00] | 1 [Reference] | 1.21 [1.02–1.42] | 1.40 [1.07–1.84] |  |  |  |
| **In the second lowest BMI category (Q2)** | | | | | |  |  |  |
| **BMI** | **Q2a: 17.94–18.25** | **Q2b: 18.26–19.57** | **Q2c: 19.58–20.50** | **Q2d: 20.51–21.30** | **Q2e: 21.31–21.43** |  |  |  |
|  | 1.45 [1.26–1.66] | 1.20 [1.11–1.30] | 1 [Reference] | 0.98 [0.90–1.06] | 1.01 [0.88–1.16] |  |  |  |
| **BRI** | **Q2a: –1.99** | **Q2b: 2.00–2.68** | **Q2c: 2.69–3.20** | **Q2d: 3.21–4.23** | **Q2e: 4.24–** |  |  |  |
|  | 0.85 [0.71–1.00] | 0.91 [0.84–0.98] | 1 [Reference] | 1.07 [0.99–1.16] | 1.29 [1.12–1.49] |  |  |  |
| **ABSI** | **Q2a: –0.73** | **Q2b: 0.74–0.79** | **Q2c: 0.80–0.83** | **Q2d: 0.84–0.90** | **Q2e: 0.91–** |  |  |  |
|  | 0.89 [0.73–1.08] | 0.92 [0.85–1.00] | 1 [Reference] | 1.18 [1.09–1.27] | 1.50 [1.31–1.72] |  |  |  |
| **In the middle BMI category (Q3)** | | | | | | |  |  |
| **BMI** | | **Q3a: 21.44–21.55** | **Q3b: 21.56–22.28** | **Q3c: 22.29–23.02** | **Q3d: 23.03–23.79** | **Q3e: 23.80–23.93** |  |  |
|  | | 1.09 [0.94–1.26] | 1.04 [0.96–1.12] | 1 [Reference] | 1.06 [0.98–1.15] | 1.07 [0.93–1.23] |  |  |
| **BRI** | | **Q3a: –2.68** | **Q3b: 2.69–3.39** | **Q3c: 3.40–3.94** | **Q3d: 3.95–5.07** | **Q3e: 5.08–** |  |  |
|  | | 0.78 [0.64–0.95] | 0.90 [0.84–0.98] | 1 [Reference] | 1.20 [1.11–1.30] | 1.48 [1.28–1.72] |  |  |
| **ABSI** | | **Q3a: –0.74** | **Q3b: 0.75–0.79** | **Q3c: 0.80–0.83** | **Q3d: 0.84–0.89** | **Q3e: 0.90–** |  |  |
|  | | 0.99 [0.82–1.19] | 0.93 [0.86–1.01] | 1 [Reference] | 1.18 [1.09–1.27] | 1.41 [1.23–1.63] |  |  |
| **In the second largest BMI category (Q4)** | | | | | | | |  |
| **BMI** | | | **Q4a: 23.94–24.07** | **Q4b: 24.08–25.02** | **Q4c: 25.03–26.27** | **Q4d: 26.28–28.43** | **Q4e: 28.44–29.03** |  |
|  | | | 0.97 [0.84–1.12] | 0.95 [0.88–1.02] | 1 [Reference] | 1.05 [0.97–1.13] | 1.15 [1.00–1.33] |  |
| **BRI** | | | **Q4a: –3.35** | **Q4b: 3.36–4.20** | **Q4c: 4.21–4.88** | **Q4d: 4.89–6.30** | **Q4e: 6.31–** |  |
|  | | | 0.74 [0.61–0.89] | 0.87 [0.80–0.94] | 1 [Reference] | 1.24 [1.14–1.34] | 1.61 [1.39–1.87] |  |
| **ABSI** | | | **Q4a: –0.74** | **Q4b: 0.75–0.79** | **Q4c: 0.80–0.82** | **Q4d: 0.83–0.88** | **Q4e: 0.89–** |  |
|  | | | 0.76 [0.63–0.93] | 0.87 [0.80–0.94] | 1 [Reference] | 1.17 [1.09–1.26] | 1.42 [1.23–1.63] |  |
| **In the largest BMI category (Q5)** | | | | | | | | |
| **BMI** | | | | **Q5a: 29.04–29.15** | **Q5b: 29.16–29.99** | **Q5c: 30.00–31.25** | **Q5d: 31.26–34.04** | **Q5e: 34.05–** |
|  | | | | 1.12 [0.78–1.59] | 1.17 [0.96–1.42] | 1 [Reference] | 1.30 [1.06–1.59] | 1.24 [0.80–1.92] |
| **BRI** | | | | **Q5a: –4.61** | **Q5b: 4.62–5.62** | **Q5c: 5.63–6.41** | **Q5d: 6.42–7.61** | **Q5e: 7.62–** |
|  | | | | 0.48 [0.27–0.83] | 0.74 [0.60–0.92] | 1 [Reference] | 1.16 [0.96–1.41] | 1.30 [0.91–1.87] |
| **ABSI** | | | | **Q5a: –0.74** | **Q5b: 0.75–0.79** | **Q5c: 0.80–0.82** | **Q5d: 0.83–0.87** | **Q5e: 0.88–** |
|  | | | | 0.74 [0.44–1.26] | 0.81 [0.65–1.01] | 1 [Reference] | 1.32 [1.10–1.59] | 1.25 [0.90–1.75] |

a. Adjusted for age, sex, lifestyle factors, and comorbidities (type 2 diabetes and cardiovascular diseases).

**Table S3. Hazard ratios of all-cause mortality according to anthropometric index quantiles in our primary and subgroup analyses stratified by sex**

| Hazard ratio (95% confidence intervals)^a^ | | | | | |  |
| --- | --- | --- | --- | --- | --- | --- |
| **Body mass index** | | | | | |  |
|  | **Q1: –17.93** | **Q2: 17.94–21.43** | **Q3: 21.44–23.93** | **Q4: 23.94–29.03** | **Q5: 29.04–** |  |
| Primary analysis | | 1.83 [1.71–1.97] | 1.21 [1.16–1.27] | 1 [Reference] | 1.00 [0.95–1.04] | 1.26 [1.16–1.37] |
| Subgroup analysis (Men) | | 2.16 [1.95–2.39] | 1.31 [1.24–1.38] | 1 [Reference] | 0.97 [0.93–1.02] | 1.22 [1.11–1.35] |
| Subgroup analysis (Women) | | 1.55 [1.39–1.72] | 1.07 [0.99–1.15] | 1 [Reference] | 1.07 [0.99–1.16] | 1.36 [1.17–1.57] |
| **Body roundness index** | | | | | | |
|  | | **Q1: –2.14** | **Q2: 2.15–3.28** | **Q3: 3.29–4.14** | **Q4: 4.15–5.99** | **Q5: 6.00–** |
| Primary analysis | | 1.34 [1.24–1.45] | 1.11 [1.07–1.16] | 1 [Reference] | 1.08 [1.04–1.13] | 1.37 [1.27–1.48] |
| Subgroup analysis (Men) | | 1.37 [1.24–1.51] | 1.13 [1.08–1.19] | 1 [Reference] | 1.10 [1.05–1.16] | 1.40 [1.24–1.59] |
| Subgroup analysis (Women) | | 1.28 [1.10–1.48] | 1.07 [0.99–1.16] | 1 [Reference] | 1.04 [0.97–1.12] | 1.32 [1.19–1.46] |
| **A body shape index** | | | | | | |
|  | | **Q1: –0.74** | **Q2: 0.75–0.79** | **Q3: 0.80–0.83** | **Q4: 0.84–0.89** | **Q5: 0.90–** |
| Primary analysis | | 0.87 [0.79–0.97] | 0.90 [0.86–0.94] | 1 [Reference] | 1.18 [1.13–1.22] | 1.47 [1.36–1.58] |
| Subgroup analysis (Men) | | 0.81 [0.71–0.92] | 0.86 [0.82–0.91] | 1 [Reference] | 1.25 [1.19–1.32] | 1.86 [1.63–2.13] |
| Subgroup analysis (Women) | | 0.96 [0.81–1.13] | 1.00 [0.91–1.09] | 1 [Reference] | 1.06 [0.98–1.14] | 1.29 [1.18–1.43] |

a. Adjusted for age, sex, lifestyle factors, and comorbidities (type 2 diabetes and cardiovascular diseases).
